# Radiological and clinical outcomes of balloon kyphoplasty for osteoporotic vertebral compression fracture in patients with rheumatoid arthritis

**DOI:** 10.1186/s13018-021-02573-5

**Published:** 2021-07-06

**Authors:** Ji Guo, Weifeng Zhai, Licheng Wei, Jianpo Zhang, Lang Jin, Hao Yan, Zheng Huang, Yongwei Jia

**Affiliations:** 1 Department of Spine Surgery, Guanghua Hospital Affiliated to Shanghai University of Traditional Chinese Medicine, No.540 Xinhua Road, Shanghai, 200052 China; 2 Shanghai Guanghua Hospital of Integrative Medicine, Shanghai, 200052 China; 3 Institute of Arthritis Research in Integrative Medicine, Shanghai Academy of Traditional Chinese Medicine, Shanghai, 200052 China

**Keywords:** Rheumatoid arthritis, Osteoporotic vertebral compression fracture, Kyphoplasty, Bone cement leakage, Adjacent vertebral fracture

## Abstract

**Background:**

This study was conducted to investigate the outcomes and complications of balloon kyphoplasty (KP) for the treatment of osteoporotic vertebral compression fracture (OVCF) in patients with rheumatoid arthritis (RA) and compare its radiological and clinical effects with OVCF patients without RA.

**Methods:**

Ninety-eight patients in the RA group with 158 fractured vertebrae and 114 patients in the control group with 150 vertebrae were involved in this study. Changes in compression rate, local kyphotic angle, visual analog scale (VAS) and Oswestry disability index (ODI) scores, conditions of bone cement leakage, refracture of the operated vertebrae, and new adjacent vertebral fractures were examined after KP. In addition, patients in the RA group were divided into different groups according to the value of erythrocyte sedimentation rate (ESR), c-reactive protein (CRP), and whether they were glucocorticoid users or not to evaluate their influence on the outcomes of KP.

**Results:**

KP procedure significantly improved the compression rate, local kyphotic angle, and VAS and ODI scores in both RA and control groups (p<0.05). Changes in compression rate and local kyphotic angle in the RA group were significantly larger than that in the control group (p<0.05), and patients with RA suffered more new adjacent vertebral fractures after KP. The outcomes and complications of KP from different ESR or CRP groups did not show significant differences. The incidence of cement leakage in RA patients with glucocorticoid use was significantly higher than those who did not take glucocorticoids. In addition, RA patients with glucocorticoid use suffered more intradiscal leakage and new adjacent vertebral fractures.

**Conclusions:**

OVCF patients with RA obtained more improvement in compression rate and local kyphotic angle after KP when compared to those without RA, but they suffered more new adjacent vertebral fractures. Intradiscal leakage and new adjacent vertebral fractures occurred more in RA patients with glucocorticoid use.

**Trial registration:**

Retrospectively registered.

## Background

RA is a heterogeneous autoimmune disease with chronic, progressive, invasive arthritis, and it manifests in 0.5–1% of the entire populatio n[1]. Osteoporosis, characterized by a reduction of bone mass per unit volume and degradation of bone microstructure, is one of the major comorbidities of RA. Some articles reported that the prevalence of osteoporosis in RA patients is approximately 30%, which is at least twice as high as those without RA [2, 3]. Nearly 50% post-menopausal women with RA had osteoporosis and RA patients in all age groups took higher risks of osteoporotic fracture than those without RA [1, 4]. Osteoporosis at epiphysis and irreversible bone destruction around the joints could occur in the early stage of RA, which followed by osteoporotic changes in the whole body and even osteoporotic fracture s[5]. Haugeberg et al. demonstrated that the longer the course of RA, the higher the incidence of osteoporotic fracture of the deformed joint s[6].

OVCF occurred in 700,000 cases in the USA each year and the clinical vertebral fracture incidence was 4.3 per 1000 person-years, but when defining fractures using radiographic screening, the data increased to 42.4 per 1000 person-year s[7, 8]. KP could effectively relieve the pain of OVCF and enable an early activity, but its outcome in OVCF patients with RA was rarely reporte d[9]. This study investigated 212 patients who suffered OVCF including 98 RA patients; clinical and radiological outcomes and complications were compared and analyzed. Besides, the influence of ESR, CRP, and whether they were glucocorticoid users on the outcomes of KP was also evaluated.

## Methods

**Fig. 1:**
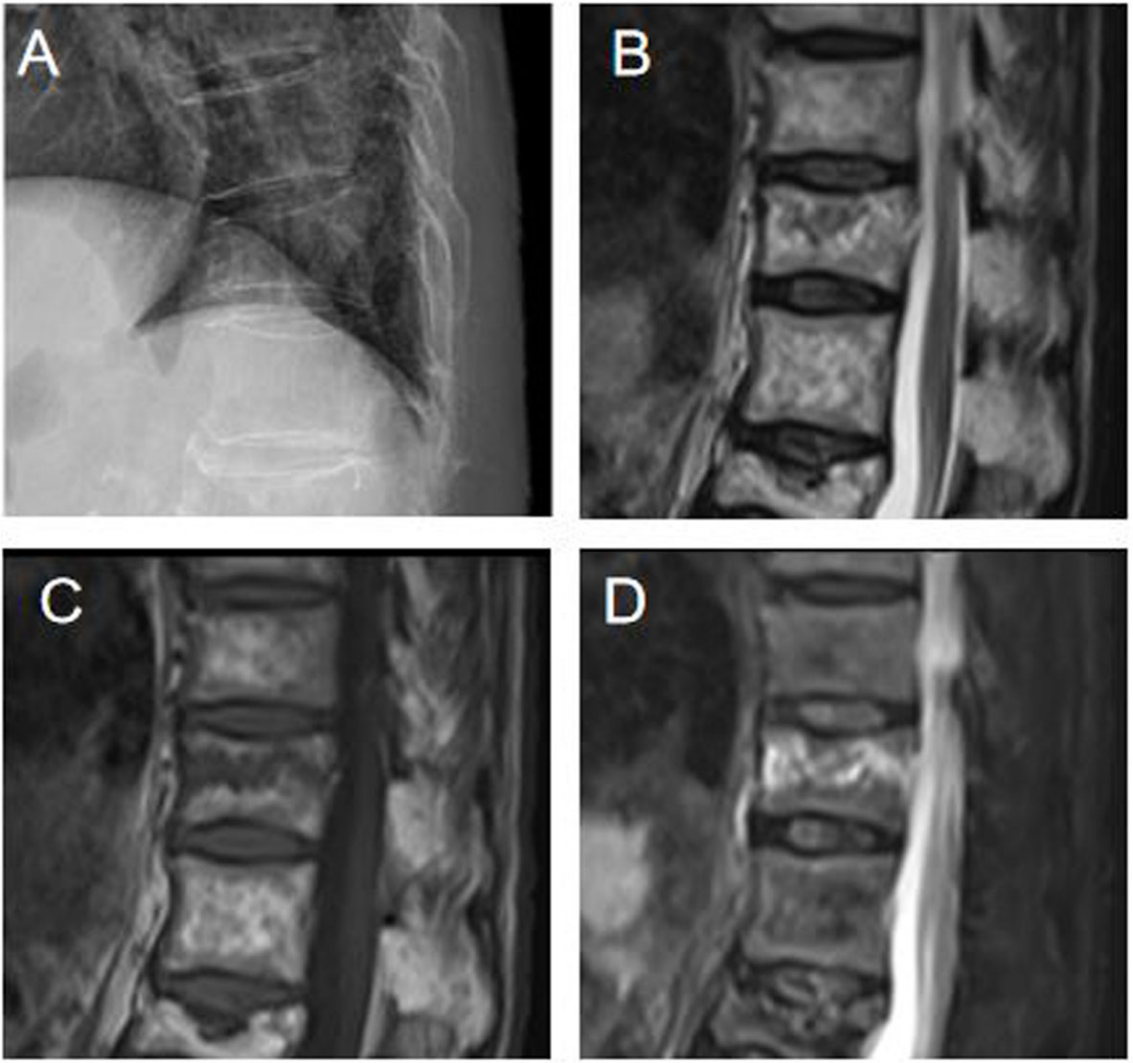
An 84-year-old female patient visited for lumbago after she slipped, and there was a T11 vertebral body fracture based on the lateral plain radiograph (**A**). A recent fracture in T2WI (**B**), T1WI (**C**), and fat suppression image (**D**) was confirmed based on the MRI

212 patients with 308 vertebral bodies diagnosed with OVCF and underwent KP from January 2014 to January 2020 were enrolled through the hospital databank. This study was approved by the Ethics Committee of Shanghai Guanghua Hospital of Integrative Medicine and conformed to the International Ethical Guidelines for Health-related Research Involving Humans issued by CIOMS. Ninety-eight patients were diagnosed with RA and osteoporosis (RA group), and the other 114 patients were osteoporotic without RA (control group). Age, gender, tobacco use, body mass index (BMI), bone mineral density (BMD), whether glucocorticoid users or not, time taken until surgery, and bone cement injection amount were collected. Besides, ESR and CRP were tested 2 days before KP. Dual-energy X-ray absorptiometry was used to test bone mineral density. Before the KP procedure, all patients were admitted to the hospital with lumbago or backache caused by slight trauma or even no obvious inducement. The total duration of pain was no longer than 6 months. Physical examination showed percussion pain in the corresponding segment of the spinous process, and there was no evidence of spinal cord or nerve injury. Thoracic and lumbar X-ray, CT scan with a three-dimensional reconstruction, and MRI were examined, and no fractures occurred in the pedicles or posterior wall of the deformed vertebral body. MRI showed a low signal of the fractured vertebral body on T1WI and a high signal of STIR sequence which indicated the presence of a fresh fracture. A representative case of OVCF was showed in Fig. 1. All patients and their families were explained the purpose, necessity, possible risks, and complications of KP, and the operation was carried out by one surgeon under general anesthesia after informed consent was signed. All included patients started to take calcitriol and calcium for anti-osteoporosis treatment the day after the KP procedure. Thoracic or lumbar X-ray and CT scan were arranged 3 days after the operation and at the 1-year follow-up.

**Fig. 2:**
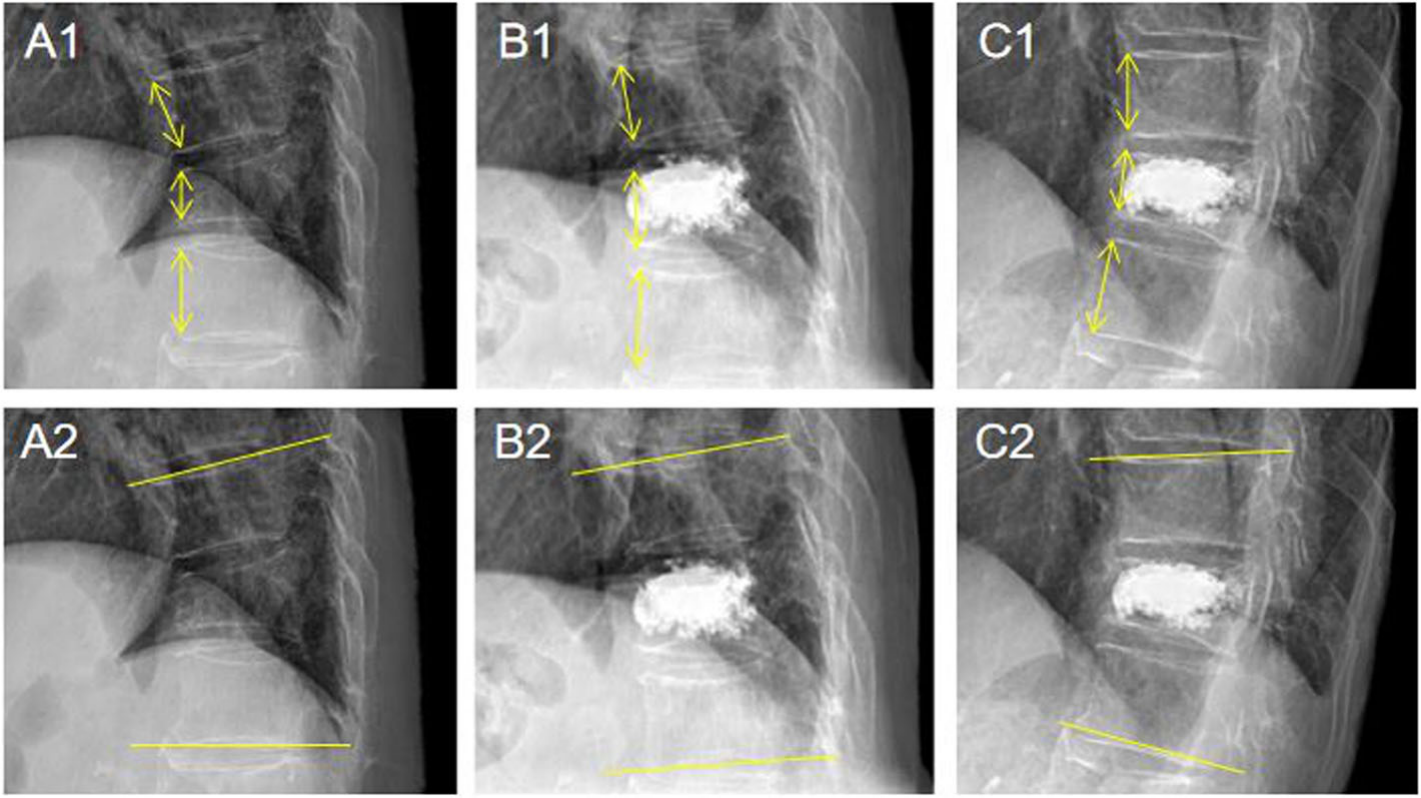
Preoperative, postoperative, and 1-year follow-up compression rate was calculated as the percentile of the height of compressed anterior vertebral body against the mean height of the adjacent top and bottom vertebral bodies (A1, B1, C1). Local kyphotic angle was examined by the angle of the superior endplate of the vertebrae above the fractured vertebra and the inferior endplate of the vertebra below the fractured vertebra (A2, B2, C2)

**Fig. 3:**
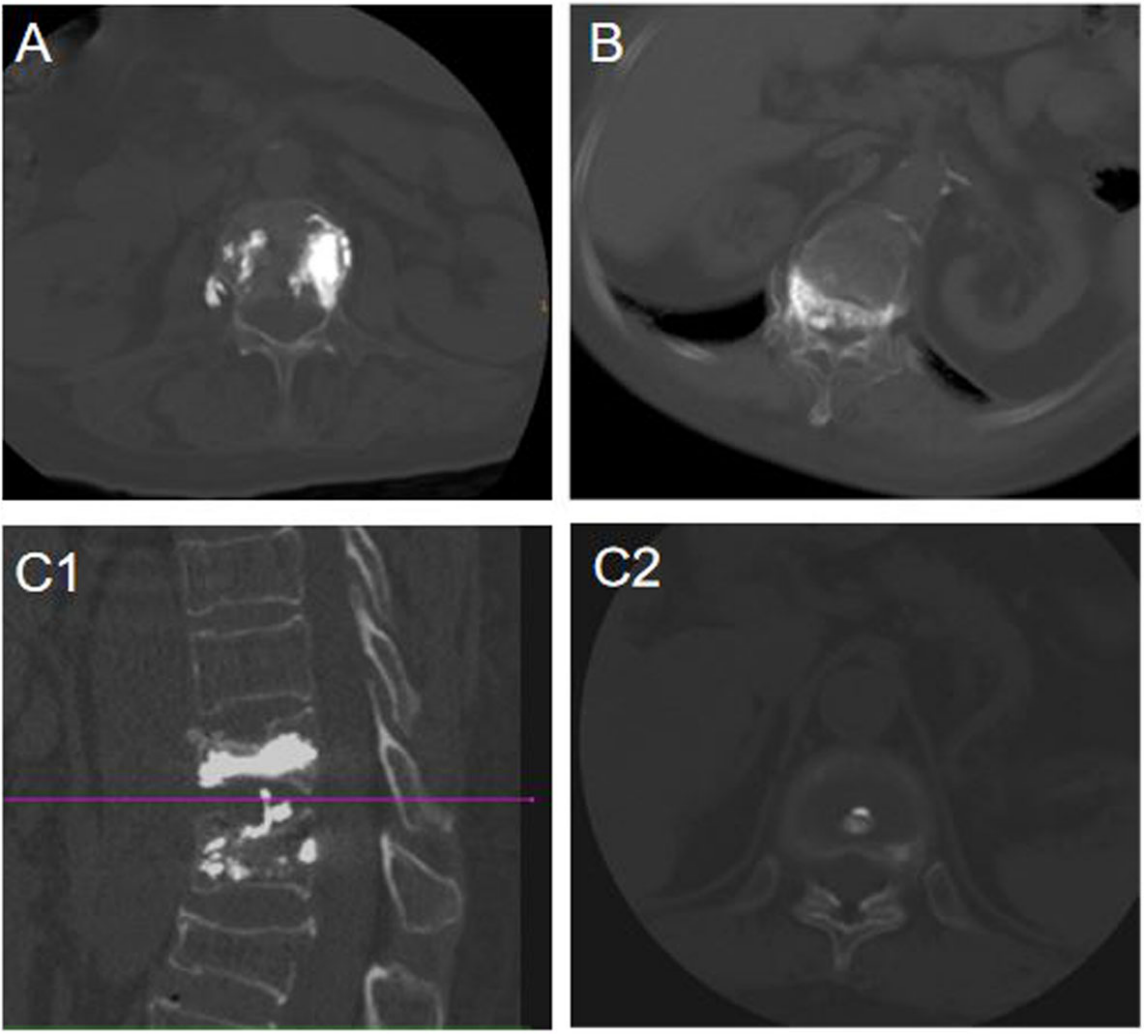
Representative images of cement leakage on a computed tomographic scan. Paravertebral cement leakage in transverse view (**A**). Intraspinal cement leakage in transverse view (**B**). Intradiscal cement leakage in sagittal and transverse view (C1, C2)

To investigate radiological outcomes, the PACS system tool was used to measure compression rate and local kyphotic angle. Compression rate was calculated as the percentile of the height of the compressed anterior vertebral body against the mean height of the anterior vertebral bodies of adjacent top and bottom vertebral bodies in a lateral radiograph. Local kyphotic angle was examined by the angle of the superior endplate of the vertebral body above the fractured vertebral body and the inferior endplate of the vertebral body below the fractured vertebral body. Figure 2 showed the measuring method for compression rate and kyphotic angle. Besides, CT scan and its three-dimensional image were used to check out the condition of cement leakage showed in Fig. 3. For clinical outcomes, VAS was used to evaluate the degree of back pain and ODI was used to assess the condition of disability. Refracture of the operated vertebral body and their new adjacent vertebral fractures were examined by MRI, and the data was collected as well.

For statistical analysis, the Student’s t test was used for the comparative analysis of continuous data. The correlation between cement leakage, refracture, and adjacent segment fracture was examined using the chi-squared and Fisher’s exact test. A paired t test was used to compare the changes in the compression rate and local kyphotic angle before and after the procedure and after a 1-year follow-up. P < 0.05 was considered statistically significant.

## Results

### Demographic data, clinical features, and complications of KP between the RA and control groups

Of all patients enrolled in the study, no serious complications such as massive hemorrhage, nerve root, spinal cord injury, postoperative pulmonary infection, and deep vein thrombosis occurred in all patients. The average blood loss was 10ml, and the operation time was 25min on average.

Ninety-eight patients aged 71.31±5.49 years old in the RA group with 158 vertebrae and 114 patients aged 73.18±4.27 years old in the control group with 150 vertebrae were involved in this study. All the demographic data and clinical characteristics were showed in Table 1. Age, gender, BMI, BMD, tobacco use, time taken from initial injury to surgery, and cement injection volume were not significantly different between the two groups. In the RA group, ESR was 35.82±24.2mm/h which is significantly higher than the control group (p<0.01). However, the value of CRP demonstrated no significant difference between the two groups (p=0.512). Sixty-five patients in the RA group were glucocorticoid users, and no patients in the control group used glucocorticoids.

**Table 1:** Demographic data, clinical features, and complications of KP between the RA and control groups

Variable	RA group	Control group	P value
Patients/vertebrae	98/ 158	114/150	
Age (year)	71.31 ± 5.49	73.18 ± 4.27	0.269
Gender (male to female)	8:90	15:99	0.244
Tobacco use	13:85	22:92	0.238
BMI (kg/m^2^)	23.2 ± 3.6	23.8 ± 3.1	0.337
BMD (g/cm^2^)	0.54 ± 0.12	0.55 ± 0.10	0.361
Glucocorticoid users	65	0	< 0.01
Time to surgery (days)	20.95 ± 18.3	16.63 ± 14.1	0.266
Cement volume (mL)	6.20 ± 1.54	6.81 ± 2.05	0.073
ESR (mm/h)	35.82 ± 24.2	20.16 ± 13.9	< 0.01
CRP (mg/dL)	10.76 ± 11.3	8.97 ± 12.5	0.512
Complications (vertebrae)			
Total cement leakage	43 (27.2%)	29 (19.3%)	0.102
Paravertebral cement leakage	18 (11.4%)	14 (9.3%)	0.554
Intradiscal cement leakage	20 (12.7%)	12 (8.0%)	0.181
Intraspinal cement leakage	5 (3.16%)	3 (2.0%)	0.521
Refracture	3 (1.9%)	2 (1.3%)	0.695
Adjacent vertebral fracture	18 (11.4%)	5 (3.3%)	< 0.01

Values are given as mean ± SD. RA indicates rheumatoid arthritis, BMI indicates body mass index, BMD indicates bone mineral density, ESR indicates erythrocyte sedimentation rate, CRP indicates c-reactive protein. Refracture indicates the operated vertebral body refractured again after KP

After the KP procedure, bone cement leakage occurred in 43 vertebral bodies in the RA group (27.2%), including 18 paravertebral leakages (11.4%), 20 intradiscal leakages (12.7%), and 5 intraspinal leakages (3.16%). No significant differences existed between the RA and control groups no matter where the leak locations were. Three operated vertebrae in the RA group refractured during the follow-up, which was not significantly different with the control group, while 18 new adjacent vertebral fractures (11.4%) occurred after KP in the RA group which was significantly higher than the control group (p<0.01).

### Radiological and clinical outcomes between the RA and control group

With regard to radiological outcomes of KP, vertebral compression rate was 59.76±13.2% before KP and it raised to 74.97±12.0% after the operation with a significant height restoration in the RA group (p<0.01). Then, it decreased to 71.32±12.2% at 1-year follow-up (p<0.01). The injured vertebrae in the control group obtained significant height restoration after KP as well (p<0.01). Besides, the local kyphotic angle significantly decreased from 7.94±5.7 to 3.93±3.8 after KP and the value was 4.17±3.8 at 1-year follow-up in the RA group (p<0.01). In the control group, the kyphotic angle was 6.70±4.9, 3.39±3.3, and 3.61±3.4 and the differences were significant as well (p<0.05). As to clinical outcomes, the VAS score significantly decreased from 8.11±0.87 to 2.02±0.68 after the KP procedure and ODI score decreased significantly from 81.12±7.32 to 24.52±4.65 in the RA group. The differences between preoperation and postoperation were significant (p<0.01). In addition, there were no significant differences in compression rate, kyphotic angle, VAS, and ODI scores between the RA group and control group at the three treatment phases, respectively (Table 2).

**Table 2:** Radiological and clinical outcomes before, after, and 1-year follow-up of KP between the RA and control groups

	Group	Preop	Postop	1-year FU	P value (Preop-Postop)	P value (Preop-1-year FU)
Compression rate (%)	RA	59.76 ± 13.2%	74.97 ± 12.0%	71.32 ± 12.2%	< 0.01	< 0.01
	Control	62.08 ± 12.6%	71.15 ± 11.8%	69.10 ± 11.9%	< 0.01	< 0.01
	P value	0.518	0.311	0.706		
Local kyphotic angle (°)	RA	7.94 ± 5.7	3.93 ± 3.8	4.17 ± 3.8	< 0.01	< 0.01
	Control	6.70 ± 4.9	3.39 ± 3.3	3.61 ± 3.4	< 0.05	< 0.05
	P value	0.380	0.553	0.268		
VAS	RA	8.11 ± 0.87	2.02 ± 0.68	2.33 ± 1.54	< 0.01	< 0.01
	Control	8.36 ± 0.69	2.21 ± 0.54	2.44 ± 1.67	< 0.01	< 0.01
	P value	0.613	0.208	0.331		
ODI	RA	81.12 ± 7.32	24.52 ± 4.65	27.31 ± 8.11	< 0.01	< 0.01
	Control	84.28 ± 6.32	21.96 ± 5.75	25.63 ± 8.21	< 0.01	< 0.01
	P value	0.587	0.364	0.418		

Values are given as mean ± SD. FU indicates follow-up, Preop indicates preoperation, Postop indicates postoperation, VAS indicates visual analog scale, and ODI indicates Oswestry disability index

### Changes in radiological and clinical outcomes between the RA and control groups

The change of vertebral compression rate was 11.56±3.8% in the RA group and 7.02±3.1% in the control group with a significant difference between them (p<0.05). The change of kyphotic angle in the RA group was 3.77±1.9, which is significantly larger than that in the control group (p<0.05). The changes in VAS score and ODI scores showed no significant differences (p=0.517, p=0.194). Therefore, changes in compression rate and local kyphotic angle in RA patients after the KP procedure were significantly larger than their changes in the control group, while changes of clinical effects were not significantly different between the two groups (Table 3).

**Table 3:** Changes of radiological and clinical outcomes between the RA and control groups

Variable	RA group	Control group	P value
Radiological outcomes			
Changes of compression rate (%)	11.56 ± 3.8%	7.02 ± 3.1%	< 0.05
Changes of local kyphotic angle (°)	3.77 ± 1.9	3.09 ± 1.6	< 0.05
Clinical outcomes			
Changes of VAS	5.78 ± 0.72	5.92 ± 0.58	0.517
Changes of ODI	53.81 ± 6.52	58.65 ± 7.13	0.194

Values are given as mean ± SD. VAS indicates visual analog scale, ODI indicates Oswestry disability index. Changes of compression rate and local kyphotic angle showed the difference of compression rate and local kyphotic angle between preoperation and 1-year follow-up; Changes of VAS and ODI showed the difference in visual analog scale and Oswestry disability index between preoperation and 1-year follow-up

### Influence of different inflammatory status on the outcome of KP

ESR and CRP are the very common indicators to show inflammatory status in RA patients. The normal range of ESR and CRP are less than 20 mm/h and 10 mg/dL, respectively. To explore the impact of different inflammatory levels on the outcome of KP in RA, we divided the 98 patients into four groups according to the value of ESR or CRP (Table 4, Table 5). No significant differences existed among groups in the aspect of age, gender, time to surgery, BMD, and number of glucocorticoid users. The condition of cement leakage, refracture of the operated vertebra, and new adjacent vertebral fractures did not show significant differences as well.

**Table 4:** Clinical features, outcomes, and complications of KP between groups of normal and elevated ESR

Variable		Normal ESR	Elevated ESR	P value
Patients/vertebrae		31/50	67/108	
Age (year)		70.5	71.7	0.356
Gender (male to female)		2:29	6:61	0.999
Time to surgery		18.45	22.1	0.284
BMD (g/cm^2^)		0.51	0.56	0.874
ESR (mm/h)		9.6	48.0	< 0.01
CRP (mg/dL)		5.45	13.22	0.043
Glucocorticoid users		21	44	0.840
Cement leakage (vertebra)		14	29	0.142
Refracture		1	2	0.999
Adjacent vertebral fracture		5	13	0.794
Radiological outcomes				
Compression rate (%)	Preop	56.88%	61.09%	0.058
	1 year FU	67.01%	73.31%	0.061
Local kyphotic angel (°)	Preop	7.58	8.11	0.080
	1 year FU	3.81	4.34	0.097
Clinical outcomes				
VAS	Preop	8.01	8.17	0.645
	1 year FU	2.18	2.40	0.416
ODI	Preop	80.06	81.61	0.561
	1 year FU	27.11	27.40	0.592

Values are given as mean ± SD. ESR indicates erythrocyte sedimentation rate, CRP indicates c-reactive protein, BMD indicates bone mineral density, and Refracture indicates the operated vertebral body refractured again after KP. Preop indicates preoperation, FU indicates follow-up, VAS indicates visual analog scale, and ODI indicates Oswestry disability index

**Table 5:** Clinical features, outcomes, and complications of KP between groups of normal and elevated CRP

Variable		Normal CRP	Elevated CRP	P value
Patients/vertebrae		26/41	72/117	
Age (year)		73.0	70.7	0.298
Gender (male to female)		2:24	6:66	0.999
Time to surgery		22.0	20.6	0.107
BMD (g/cm^2^)		0.53	0.54	0.912
ESR (mm/h)		31.0	37.6	0.114
CRP (mg/dL)		1.65	14.05	< 0.01
Glucocorticoid users		20	45	0.230
Cement leakage (vertebra)		11 (26.8%)	32 (27.3%)	0.947
Refracture		1	2	0.999
Adjacent vertebral fracture		4	14	0.999
Radiological outcomes				
Compression rate (%)	Preop	62.28%	58.89%	0.450
	1 year FU	73.21%	70.66%	0.281
Local kyphotic angel (°)	Preop	7.95	7.94	0.692
	1 year FU	4.25	4.14	0.074
Clinical outcomes				
VAS	Preop	8.05	8.13	0.563
	1 year FU	2.27	2.35	0.585
ODI	Preop	80.16	81.47	0.412
	1 year FU	27.02	27.41	0.493

Values are given as mean ± SD. ESR indicates erythrocyte sedimentation rate, CRP indicates c-reactive protein, BMD indicates bone mineral density, and Refracture indicates the operated vertebral body refractured again after KP. Preop indicates preoperation, FU indicates follow-up, VAS indicates visual analog scale, and ODI indicates Oswestry disability index

At the 1-year follow-up, vertebral compression rates were all increased and there were no significant differences among the groups no matter before or 1 year after the procedure. Local kyphotic angles were all decreased as well. With regard to clinical outcomes, VAS and ODI scores significantly declined in all groups and no differences existed among them.

### Outcomes and complications of KP between glucocorticoid users and non-glucocorticoid users in RA

Ninety-eight patients in the RA group were divided into 2 groups according to whether they were glucocorticoid users (Table 6). The average BMD of glucocorticoid users was significantly lower than the other group (p=0.041). The percentage of cement leakage in glucocorticoid users was significantly higher than the other group (p<0.01). In detail, only intradiscal leakage showed a significant difference between the two groups (p=0.027). Only 1 operated vertebra in non-glucocorticoid users refractured during the follow-up, which was significantly less than the adjacent vertebral fractures in glucocorticoid users (p=0.044). As to the radiological and clinical outcomes, compression rate, kyphotic angle, VAS, and ODI scores significantly changed at the 1-year follow-up after KP in both groups, but no significant differences existed between the two groups.

**Table 6:** Outcomes and complications of KP between glucocorticoid users and non-glucocorticoid users in RA

Variable		Glucocorticoid users	Non-glucocorticoid users	P value
Glucocorticoid users/vertebrae		65/116	33/42	
Age (year)		72.1	69.8	0.246
Gender (male to female)		5:60	3:30	0.999
BMD (g/cm^2^)		0.51	0.60	0.041
Time to surgery		21.24	20.38	0.382
ESR (mm/h)		33.9	39.6	0.106
CRP (mg/dL)		9.0	13.95	0.061
Cement volume (mL)		6.13	6.34	0.369
Cement leakage (vertebra)		38 (32.8%)	5 (11.9%)	0.009
Paravertebral leakage		15	3	0.404
Intradiscal leakage		19	1	0.027
Intraspinal leakage		3	2	0.609
Refracture		2	1	0.999
Adjacent vertebral fracture		17	1	0.044
Radiological outcomes				
Compression rate (%)	Preop	58.57%	63.05%	0.185
	1 year FU	69.97%	75.05%	0.102
Local kyphotic angel (°)	Preop	7.86	8.16	0.344
	1 year FU	3.98	4.69	0.074
Clinical outcomes				
VAS	Preop	8.13	8.07	0.652
	1 year FU	2.31	2.37	0.564
ODI	Preop	81.90	79.58	0.449
	1 year FU	26.78	28.35	0.506

Values are given as mean ± SD. BMD indicates bone mineral density, ESR indicates erythrocyte sedimentation rate, CRP indicates c-reactive protein, and Refracture indicates the operated vertebral body refractured again after KP. Preop indicates preoperation, FU indicates follow-up, VAS indicates visual analog scale, and ODI indicates Oswestry disability index

## Discussion

RA is a systemic autoimmune disease featured by continuous synovitis and invasive damage of the joints. It is well known that rheumatoid arthritis progresses with the destruction of bone microstructure and loss of bone mass, thus increasing the incidence of osteoporotic fracture. Lee et al. reported that the incidence of osteoporosis in patients with RA was 22.1%, which was approximately twice that in healthy people (11.4% )[10]. Tong and Ghazi separately demonstrated the incidence of OVCF in RA patients was 21.7% and 19.2%, which was almost 5 times higher than the healthy group [5, 11]. Some studies suggested that osteoporosis in RA patients and primary osteoporosis shared similar risk factors, including older age, female sex, and underweight [12]. However, the risk of osteoporosis in RA patients was related to the disease itself as well; there was a positive correlation with higher disease activity, longer disease course, and less participation in daily activities.

Conservative management strategies of OVCF included bed rest, analgesia, and physical therapy [13]. A long-term bed rest would furtherly aggravate osteoporosis and lead to bedsore, lung infection, depression, and other complications with an increased mortality in the elderly. Besides, the height of the vertebral body lost and local kyphosis rose is due to worse compliance [14]. Papanastassiou et al. reviewed that KP and vertebroplasty (VP) showed an improved pain-reducing effect over conservative management and KP demonstrated enhanced results for quality of life improvement over VP [15–17]. KP could restore vertebral height through balloon inflation and dynamic fracture mobility supported by lots of clinical researc h[18–21]. However, few articles reported the effects of KP in RA patients with OVCF. In our study, no matter in the RA or control group, vertebral compression rate significantly increased and local kyphotic angles significantly decreased after KP. VAS and ODI scores were also significantly decreased after the procedure. In addition, the changes in compression rate and kyphotic angle in the RA group were significantly larger than the control group. The higher vertebral compression rate after KP indirectly indicated weaker bone quality in patients with RA. Chronic inflammation in RA can impact bone metabolism, thus leading to abnormal bone resorption and impaired bone formation [22]. This might be also associated with glucocorticoid, the most commonly prescribed medicine for RA. Though disease activity and inflammation-related bone erosion could be alleviated, glucocorticoid could also inhibit the activity of osteoblasts and promote osteoclasts mediated bone resorption and bone matrix decomposition, leading to a rapid decline in bone mineral densit y[20, 23–25]. Kumagai revealed that glucocorticoids could induce the extinction of osteocytes, leading to the destruction of bone qualit y[26].

Cement leakage is one of the most common complications of KP which is related to the viscosity and volume of injected cement, the extent of the compression, pressure and speed of injection, and other factor s[27, 28]. The leakage locations include nerve root canal, intraspinal epidural space, paravertebral soft tissue, intervertebral disc, paravertebral venous plexus, and puncture needle channels. During the KP procedure, the incidence of bone cement leakage is from 9.6 to 33 %[29–32]. In our study, the percentage of cement leakage of the RA and control group was 27.2% and 19.3% respectively, and the difference between them was not statistically significant. Besides, the value of ESR and CRP did not impact the leakage conditions as well. Glucocorticoid users were more likely to suffer cement leakage than those who did not take glucocorticoids. In specific, intradiscal leakage occurred more in RA patients with glucocorticoid use, and no statistical differences existed in paravertebral and intraspinal leakage between the two groups. The reason might be that glucocorticoid treatment impacts bone formation and resorption, resulting in a weak cortical bone and endplate of vertebrae, thus increasing cement leakage, especially in intervertebral discs.

According to the results, OVCF patients with RA took more risks in suffering new vertebral fractures than the control group. Klazen and Shi suggested the natural progression of osteoporosis was the main reason for the new fractures [33, 34]. While Hulme et al. proposed that new adjacent vertebral fractures did not occur as long as bone cement did not reach the vertebral endplate s[35]. Harrop et al. revealed that the incidence of symptomatic refractures in patients receiving oral steroid therapy at their initial vertebral cement injection was almost twice of those primary osteoporosis patients [36]. Similarly, we also found that new adjacent fractures occurred more in RA patients with glucocorticoid use than in the control group. This might be related to the high incidence of discal cement leakage after KP in these patients. Bone cement leakage may aggravate degeneration of the intervertebral disc and furtherly accelerate the degeneration of adjacent vertebral bodies. During cement extravasation, the heat produced by solidification resulted in irreversible damage of the bone, blood vessels, and even endplates. At the same time, the solidified bone cement, injured vertebra, and intervertebral disc formed a conjunct segment of high stiffness, which not only limited the vertebral activity, but also reduced the buffering effect for stress, increasing the risk of adjacent vertebral fractures. Besides, the leaking cement in discs may impact the absorption of nutrients and the metabolism of discs, aggravating the damage of intervertebral discs and adjacent vertebrae. Liu et al. also believed that the occurrence of bone cement leakage lost the elastic protection of the intervertebral disc, increased the stress load of the adjacent vertebral body, and became the predilection site of vertebral fracture after operation [37]. Lin et al. stated that the risk of adjacent fracture elevated four-fold when cement leakage took place within the intervertebral disc [38].

Though this study revealed some characteristics of KP in RA patients who suffered OVCF, there were several limitations as well. First of all, it was a retrospective study without analysis of sagittal imbalance of the spine. Besides, there was no quantitative comparison on the dosage of glucocorticoid use in RA patients due to their different frequency and dose adjustment of glucocorticoids. Since this was short-term follow-up research, further study of a long-term clinical study with more subjects and quantitative design is required for a better exploration of OVCF in RA patients.

## Conclusions

KP procedure was effective for OVCF patients with or without RA, for reducing kyphotic angle, relieving pain, and restoring vertebral body height and spinal function. Compared to the control group, RA patients received more improvement in compression rate and local kyphotic angle after the operation, but they had more risks in adjacent vertebral fractures, whereas the change of VAS and ODI scores were not different between patients with or without RA. In addition, ESR, CRP, and whether with glucocorticoid use did not significantly affect the radiological and clinical outcomes, but new adjacent vertebral fractures and cement leakage, especially intradiscal leakage occurred more in RA patients with glucocorticoid use.

## Data Availability

The datasets used and/or analyzed during the current study are available from the corresponding author on reasonable request.

## Abbreviations

KP: Kyphoplasty
OVCF: Osteoporotic vertebral compression fracture
RA: Rheumatoid arthritis
ESR: Erythrocyte sedimentation rate
CRP: C-reactive protein
VAS: Visual analog scale
ODI: Oswestry disability index
VP: Vertebroplasty
Preop: Preoperation
Postop: Postoperation
FU: Follow-up

## References

[1] Kvien TK, Haugeberg G, Uhlig T, Falch JA, Halse JI, Lems WF, Dijkmans BA, Woolf AD (2000). Data driven attempt to create a clinical algorithm for identification of women with rheumatoid arthritis at high risk of osteoporosis. Ann Rheum Dis.

[2] Hauser B, Riches PL, Wilson JF, Horne AE, Ralston SH (2014). Prevalence and clinical prediction of osteoporosis in a contemporary cohort of patients with rheumatoid arthritis. Rheumatol Oxf Engl.

[3] Haugeberg G, Uhlig T, Falch JA, Halse JI, Kvien TK (2000). Bone mineral density and frequency of osteoporosis in female patients with rheumatoid arthritis: results from 394 patients in the Oslo County Rheumatoid Arthritis Register. Arthritis Rheum.

[4] Lee JH, Sung YK, Choi CB, Cho SK, Bang SY, Choe JY, Hong SJ, Jun JB, Kim TH, Lee J, Lee HS, Yoo DH, Yoon BY, Bae SC (2016). The frequency of and risk factors for osteoporosis in Korean patients with rheumatoid arthritis. BMC Musculoskelet Disord.

[5] Tong JJ, Xu SQ, Zong HX, Pan MJ, Teng YZ, Xu JH (2020). Prevalence and risk factors associated with vertebral osteoporotic fractures in patients with rheumatoid arthritis. Clin Rheumatol.

[6] Haugeberg G, Green MJ, Quinn MA, Marzo-Ortega H, Proudman S, Karim Z, Wakefield RJ, Conaghan PG, Stewart S, Emery P (2006). Hand bone loss in early undifferentiated arthritis: evaluating bone mineral density loss before the development of rheumatoid arthritis. Ann Rheum Dis.

[7] Mirovsky Y, Anekstein Y, Shalmon E, Blankstein A, Peer A (2006). Intradiscal cement leak following percutaneous vertebroplasty. Spine.

[8] Jin S, Hsieh E, Peng L, Yu C, Wang Y, Wu C, Wang Q, Li M, Zeng X (2018). Incidence of fractures among patients with rheumatoid arthritis: a systematic review and meta-analysis. Osteoporos Int.

[9] Lee DG, Park CK, Park CJ (2015). Analysis of risk factors causing new symptomatic vertebral compression fractures after percutaneous vertebroplasty for painful osteoporotic vertebral compression fractures: a 4-year follow-up. J Spinal Disord Tech.

[10] Lee SG, Park YE, Park SH, Kim TK, Choi HJ, Lee SJ, Kim SI, Lee SH, Kim GT, Lee JW, Lee JH, Baek SH (2012). Increased frequency of osteoporosis and BMD below the expected range for age among South Korean women with rheumatoid arthritis. Int J Rheum Dis.

[11] Ghazi M, Kolta S, Briot K, Fechtenbaum J, Paternotte S, Roux C (2012). Prevalence of vertebral fractures in patients with rheumatoid arthritis: revisiting the role of glucocorticoids. Osteoporos Int.

[12] Soen S, Fukunaga M, Sugimoto T (2013). Diagnostic criteria for primary osteoporosis: year 2012 revision. J Bone Miner Metab.

[13] Ito M, Harada A, Nakano T, Kuratsu S, Deguchi M, Sueyoshi Y, Machida M, Yonezawa Y, Matsuyama Y, Wakao N (2010). Retrospective multicenter study of surgical treatments for osteoporotic vertebral fractures. J Orthop Sci.

[14] Tanaka Y, Ohira T (2018). Mechanisms and therapeutic targets for bone damage in rheumatoid arthritis, in particular the RANKRANKL system. Curr Opin Pharmacol.

[15] Papanastassiou ID, Phillips FM, Van Meirhaeghe J (2012). Comparing effects of kyphoplasty, vertebroplasty, and non-surgical management in a systematic review of randomized and non-randomized controlled studies. Eur Spine J.

[16] Denaro V, Longo UG, Maffulli N, Denaro L (2009). Vertebroplasty and kyphoplasty. Clin Cases Miner Bone Metab.

[17] Longo UG, Loppini M, Denaro L, Brandi ML, Maffulli N, Denaro V (2010). The effectiveness and safety of vertebroplasty for osteoporotic vertebral compression fractures. A double blind, prospective, randomized, controlled study. Clin Cases Miner Bone Metab.

[18] Grohs JG, Matzner M, Trieb K, Krepler P (2005). Minimal invasive stabilization of osteoporotic vertebral fractures: a prospective nonrandomized comparison of vertebroplasty and balloon kyphoplasty. J Spinal Disord Tech.

[19] Lovi A, Teli M, Ortolina A, Costa F, Fornari M, Brayda-Bruno M (2009). Vertebroplasty and kyphoplasty: complementary techniques for the treatment of painful osteoporotic vertebral compression fractures. A prospective non-randomised study on 154 patients. Eur Spine J.

[20] Redlich K, Smolen JS (2012). Inflammatory bone loss: pathogenesis and therapeutic intervention. Nat Rev Drug Discov.

[21] Jacobs JW, de Nijs RN, Lems WF, Geusens PP, Laan RF, Huisman AM, Algra A, Buskens E, Hofbauer LC, Oostveen AC, Bruyn GA, Dijkmans BA, Bijlsma JW (2007). Prevention of glucocorticoid induced osteoporosis with alendronate or alfacalcidol: relations of change in bone mineral density, bone markers, and calcium homeostasis. J Rheumatol.

[22] Mundy GR (2007). Osteoporosis and inflammation. Nutr Rev.

[23] Hartmann K, Koenen M, Schauer S, Wittig-Blaich S, Ahmad M, Baschant U, Tuckermann JP (2016). Molecular actions of glucocorticoids in cartilage and bone during health, disease, and steroid therapy. Physiol Rev.

[24] Kumagai S, Kawano S, Atsumi T, Inokuma S, Okada Y, Kanai Y, Kaburaki J, Kameda H, Suwa A, Hagiyama H, Hirohata S, Makino H, Hashimoto H (2005). Vertebral fracture and bone mineral density in women receiving high dose glucocorticoids for treatment of autoimmune diseases. J Rheumatol.

[25] Zhang ZF, Huang H, Chen S, Liu DH, Feng YH, Xie CL, Jiao F (2018). Comparison of high and low viscosity cement in the treatment of vertebral compression fractures: a systematic review and meta-analysis. Medicine.

[26] Wang B, Zhao CP, Song LX (2018). Balloon kyphoplasty versus percutaneous vertebroplasty for osteoporotic vertebral compression fracture: a meta-analysis and systematic review. Orthop Surg Res.

[27] Zhai WF, Jia YW, Wang JJ, Cheng L, Zeng Z, Yu Y, Chen L (2015). The clinical effect of percutaneous kyphoplasty for the treatment of multiple osteoporotic vertebral compression fractures and the prevention of new vertebral fractures. Int J Clin Exp Med.

[28] Heini PF, Orler R (2004). Kyphoplasty for treatment of osteoporotic vertebral fractures. Eur Spine J.

[29] Ledlie JT, Renfro MB (2006). Kyphoplasty treatment of vertebral fractures: 2-year outcomes show sustained benefits. Spine.

[30] Zhai WF, Jia YW, Wang JJ, Cheng L (2015). The clinical application and efficacy of percutaneous kyphoplasty via unilateral pedicular approach guided by CT image measurement. Int J Clin Exp Med.

[31] Klazen CA, Venmans A, de Vries J (2010). Percutaneous vertebroplasty is not a risk factor for new osteoporotic compression fractures: results from VERTOS II. AJNR Am J Neuroradiol.

[32] Shi MM, Cai XZ, Lin T, Wang W, Yan SG (2012). Is there really no benefit of vertebroplasty for osteoporotic vertebral fractures? A meta-analysis. Clin Orthop Relat Res.

[33] Hulme PA, Boyd SK, Heini PF, Ferguson SJ (2009). Differences in endplate deformation of the adjacent and augmented vertebra following cement augmentation. Eur Spine J.

[34] Harrop JS, Prpa B, Reinhardt MK, Lieberman I (2004). Primary and secondary osteoporosis’ incidence of subsequent vertebral compression fractures after kyphoplasty. Spine.

[35] Liu T, Zhe L, Su Q (2017). Cement leakage in osteoporotic vertebral compression fractures with cortical defect using high-viscosity bone cement during unilateral percutaneous kyphoplasty surgery. Medicine..

[36] Lin EP, Ekholm S, Hiwatashi A, Westesson PL (2004). Vertebroplasty: cement leakage into the disc increases the risk of new fracture of adjacent vertebral body. Am J Neuroradiol.

[37] Voggenreiter G (2005). Balloon kyphoplasty is effective in deformity correction of osteoporotic vertebral compression fractures. Spine.

[38] Liu JT, Liao WJ, Tan WC, Lee JK, Liu CH, Chen YH, Lin TB (2010). Balloon kyphoplasty versus vertebroplasty for treatment of osteoporotic vertebral compression fracture: a prospective, comparative, and randomized clinical study. Osteoporos Int.

